# Integrative Omics Analysis Reveals a CD206^+^ Macrophage/Dendritic Cell–CD155–TIGIT^+^ Regulatory T Cell Axis as a Protective Immune Checkpoint Pathway in Atherosclerosis Progression

**DOI:** 10.1002/dni2.70016

**Published:** 2026-09-17

**Authors:** Ying Shao, Fatma Saaoud, Mohammed Ben Issa, Keman Xu, Yifan Lu, Shih-Yu Hung, Juanjuan Liu, Bo Yuan, Juncheng Wei, Sheng Wu, Sadia Mohsin, Zhengjie Zhou, Beata Kosmider, Laisel Martinez, Roberto Vazquez-Padron, Hong Wang, Xiaofeng Yang

**Affiliations:** 1 Lemole Center for Integrated Lymphatics and Vascular Research, Philadelphia, Pennsylvania, USA; 2 Centers for Metabolic Disease Research and Thrombosis Research, Philadelphia, Pennsylvania, USA; 3 Department of Cardiovascular Sciences, Aging+ Cardiovascular Discovery Center, Philadelphia, Pennsylvania, USA; 4 Center for Inflammation, Translational and Clinical Lung Research, Lewis Katz School of Medicine, Temple University, Philadelphia, Pennsylvania, USA; 5 DeWitt Daughtry Family Department of Surgery, Leonard M. Miller School of Medicine, University of Miami, Miami, Florida, USA

**Keywords:** atherosclerosis, CD155, CD206^+^ macrophages (MØs)/dendritic cells (DCs), immune checkpoints (ICs), TIGIT

## Abstract

RNA sequencing (RNA-seq) analysis revealed transcriptional features indicative of sustained immunosuppression within regulatory T cells (Tregs) isolated from atherosclerotic tissues, highlighting the critical contribution of immune checkpoint (ICP) receptor–ligand interactions to atherosclerosis progression. To comprehensively characterize the roles of ICP signaling in this context, we integrated transcriptomic profiling, quantitative RT-PCR, and flow cytometry analysis, yielding several key findings: (1) Multiomics integration revealed global transcriptomic alterations in ICP receptors and ligands across diverse tissues, immune cell types, and disease stages in both human and murine models of atherosclerosis; (2) Several ICP components demonstrated functional relevance, including dual-function ligand CD155 (PVR); inhibitory ligands Lgals3 and Itgb1; stimulatory ligands Tnfrsf9 and CD48; and inhibitory receptors Havcr2 and Lair1. These molecules act as microenvironmental sensors, dynamically responding to atherosclerotic cues; (3) Experimental validation confirmed upregulation of the CD155-TIGIT axis within immune cells of atherosclerotic plaques. Notably, CD155 expression positively correlated with CD206 expression in both CD11b^+^CD11c^+^ and CD11b^−^CD11c^+^ cells, irrespective of tissue context; and (4) The CD206+ macrophages/DCs–CD155–TIGIT^+^ Treg axis emerged as a potential ICP-mediated pathway that confers immunoregulatory protection against atherosclerosis progression. Collectively, these findings uncover novel immunomodulatory roles for ICP ligands in atherosclerosis, expanding their known functions beyond adaptive immune regulation to include modulation of innate immune responses within atherosclerotic lesions.

## 1 | Introduction

Immune checkpoints (ICPs) are plasma membrane proteins with immunological activity that maintain self-tolerance, regulate immune responses, and prevent tissue damage [1]. Immune checkpoint signaling is initiated when the T cell antigen receptor (TCR) recognizes antigen epitopes presented by major histocompatibility complex II on antigen-presenting cells (APCs), serving as an additional mechanism for T cell activation or exhaustion [2, 3]. Classic ICPs include coinhibitory receptors, which suppress T cell activity, and costimulatory receptors, which enhance T cell activation. The balance between these opposing signals governs overall ICP functions [4]. Therapeutically, targeting inhibitory pathways of prototypic ICPs such as PD-1/PD-L1 or CD80/CD86-CTLA-4 has revolutionized cancer immunotherapy [5, 6], and emerging evidence implicates the roles of ICPs in cardiovascular diseases (CVDs) as well [7, 8].

Inflammation and immune modulation play central roles in CVDs, as exemplified by Canakinumab, an anti-inflammatory monoclonal antibody targeting interleukin-1β (IL-1β), which reduces atherosclerotic events [9]. Immune cells, including T cells [10–13] and APCs, respond to inflammatory cues and contribute to CVD initiation and progression [14, 15]. Functional studies in mouse models and human patients indicate that ICP molecules modulate risk and progression of multiple CVDs, including vein graft disease [16], graft arterial disease [17], coronary heart disease, ischemic stroke [18, 19], myocarditis [20], cardiomyopathy [21], heart failure [22], and atherosclerosis [23–26]. Immune checkpoint receptors and ligands exhibit diverse and context-dependent roles. For example, CD47 interacts with the inhibitory receptor signal regulator protein alpha (SIRPα) [27]. Anti-CD47 treatment reduces vascular inflammation and atherosclerosis in apolipoprotein E (ApoE)^−/−^ mice by restoring efferocytosis [28, 29], whereas selective inhibition of SIRPα signaling more effectively decreases necrotic core size [30]. Both CD47 and SIRPα are upregulated in human atherosclerotic arteries, predominantly in CD68+ macrophage areas. Myeloid-specific SIRPα deficiency promotes cholesterol efflux, induces an anti-inflammatory M2 macrophage phenotype, and suppresses necrotic core formation, whereas myeloid CD47 loss impairs cholesterol efflux and worsens lesions, highlighting the specificity of the myeloid-SIRPα/T cell-CD47 axis. Another example is CD40 ligand (CD40L) on T cells, which activates APCs upon binding CD40. Anti-CD40L therapy reduces early atherosclerosis in Ldlr^−/−^ mice by decreasing macrophage and T cell infiltration [31] and limits progression of established atherosclerotic lesions [32]. However, in CD40^−/−^/ApoE^−/−^ mice, a significant difference is observed only in advanced plaques, not in initial plaques, compared to ApoE^−/−^ controls [33]. Cell type-specific knockout studies further reveal that T cell CD40L deficiency reduces plaque burden and promotes plaque stability, whereas platelet CD40L loss primarily affects thrombus formation without altering plaque development [34]. These outcomes emphasize the need for a comprehensive framework that accounts for disease stage and cell type specificity in ICP-mediated regulation of CVDs.

Regulatory T cells (Tregs) exert atheroprotective effects through anti-inflammatory cytokines (IL-10, TGF-β), inhibition of Th1/Th17 response, and downregulation of dendritic cells (DCs) antigen presentation [35]. Inhibitory ICPs are critical for these functions, although their contributions vary [36, 37]. For instance, CTLA-4 knock-in in atherosclerotic mice enhanced Treg suppressive function and reduces lesion formation without altering CD4^+^Foxp3^+^ Tregs proportions in the aortic root plaques [24], whereas PD-1 knockout leads to accumulation of activated T cells, including Tregs, without affecting suppressive activity [38]. Our previous study identified additional inhibitory receptors upregulated in CD4^+^Foxp3^+^ Tregs from atherosclerotic mice [15], underscoring the need to elucidate their functional roles and the relevant ligand–receptor axes. Investigating ICP receptor–ligand interactions between Tregs and APCs will further clarify Treg-mediated suppression in atherosclerosis.

Despite advances in tumor immunology and growing understanding of ICPs in CVDs, key knowledge gaps remain: (1) the expressions of 58 ICP receptors–ligands pairs (86 genes in total) in the human and murine aortas with atherosclerosis are poorly characterized; (2) ICP expressions in non-APCs cell types within the aorta are largely unknown; (3) the mutual regulations of ICP receptors and ligands in atherosclerotic regulatory T cells and their target cells are not well understood; and (4) ICP-based immune cell biomarkers require further exploration. By integrating -Omics data with experimental validation (Figure 1), our study addresses these gaps and provide novel insights into aortic diseases and ICP-mediated immune regulation.

## 2 | Materials and Methods

### 2.1 | Ingenuity Canonical Pathway Analysis

A total of 2942 differentially expressed genes (DEGs; *p* < 0.05) identified by RNA sequencing (RNA-seq) analysis of CD4^+^ Foxp3^+^ Tregs from atherosclerotic versus control mice were uploaded into Ingenuity Pathway Analysis (IPA) (Qiagen, Redwood City, CA). Canonical pathway enrichment was performed using the Ingenuity Knowledge Base. A bubble plot of significantly enriched canonical pathways was generated using RStudio (https://en.wikipedia.org/wiki/RStudio).

### 2.2 | Transcriptomic Analyses of ICPs in Hyperlipidemia and Atherosclerosis Mouse Models

Expression profiles of ICP genes in the atherosclerotic mice and in vitro studies were collected from 37 published transcriptomic datasets obtained from the NIH-NCBI Gene Expression Omnibus database (https://www.ncbi.nlm.nih.gov/gds/), as we previously described [39–43]. The datasets IDs are listed in Table S1, along with one set of our own RNA-sequencing results. DEGs were identified in each dataset using a threshold of *p* < 0.05. To minimize bias caused by differences in experimental design and sequencing platforms, log_2_ fold change (log_2_FC) values from each comparison were used to provide an overall visualization of the direction and magnitude of expression changes across independent datasets.

### 2.3 | Single-Cell Transcriptome Analysis of ICP Gene Distribution in the Atherosclerotic Aorta

Single-cell RNA-seq data were retrieved and processed from three publicly available datasets [44]. Differential gene expressions within clusters were calculated as the log2FC between the percentages of cells expressing a given gene within the cluster versus outside the cluster. Heatmaps were generated to visualize the cell-type distribution of ICP-related gene expression.

### 2.4 | Animals

All mice were on C57BL/6 background. Wide-type (WT), atherogenic apolipoprotein E deficient (ApoE^−/−^; stock no. 002052), and *Foxp3*^*YFP-Cre*^ (stock no. 016959), Foxp3-GFP mice (stock no. 006772) were purchased from the Jackson Laboratory (Bar Harbor, ME). Foxp3^YFP−Cre^/ApoE^−/−^ (yellow fluorescent protein (YFP)/ApoE^−/−^), Foxp3^GFP^/ApoE^−/−^ mice were cross-bred in our laboratory. Only male mice were used for experiments. Mice were maintained on a standard chow diet (CD) until 8 weeks of age. For atherosclerosis induction, the mice were switched to a high-fat diet (HFD) (AIN-76A Western Diet, TestDiet, CA) containing 0.2% (w/w) cholesterol and 21.2% (w/w) fat starting at 8 weeks of age. All animal studies were conducted in accordance with the Institutional Animal Care and Use Committee Guidelines and approved protocols of Temple University Lewis Katz School of Medicine.

### 2.5 | RNA Isolation and Quantitative PCR (qPCR)

RNAs from flash-frozen aorta were isolated using the miRNeasy Mini Kit (Qiagen, 217004). The cDNA was synthesized using High-Capacity cDNA Reverse Transcription Kit (Applied Biosystems, 4368814), and Quantitative PCR (qPCR) was performed with iTaq Universal SYBR Green Supermix (Bio-Rad). Results were calculated using the ΔΔCt method relative to the reference control gene *β*-actin. Sequences of mouse primer *Pvr* are Forward 5′-GTCTTTGTCCTCGCTGTAGTC-3′, Reverse 5′-GGCTCCATGTTCAGTCTACAG-3′, primer *Actin* are Forward 5′-ACCTTCTACAATGAGCTGCG-3′, and Reverse 5′-CTGGATGGCTACGTACATGG-3'.

### 2.6 | Cell Preparation for Flow Cytometry

#### 2.6.1 | Blood

Approximately 300 μL of blood was collected via cardiac puncture immediately after euthanasia and mixed with 1 mL phosphate-buffered saline (PBS) and 5 mL ammonium-chloride-potassium (ACK) lysing buffer. After 5 minutes, 8 mL of flow cytometry (FACS) buffer was added to terminate cell lysis. Cells were centrifuged at 350 × *g* for 5 min at 4°C, the supernatant was removed, and pellets were resuspended in ice-cold FACS buffer.

#### 2.6.2 | Kidney and Spleen

Freshly isolated kidneys were gently homogenized between frosted glass slides, rinsed with FACS buffer, and transferred into 15 mL conical tubes. Samples were centrifuged at 350 × *g* for 5 minutes at 4°C. Splenic cell pellets were lysed with 1 mL ACK buffer for 5 minutes and then washed with FACS buffer. After centrifugation, cells were filtered through a 40 μm strainer, and cell concentrations were adjusted to 1 × 10^6^–5 × 10^6^ cells/mL.

#### 2.6.3 | Lungs

Lungs were perfused with 10 mL PBS via cardiac puncture, washed twice, minced into ~2 mm pieces, and digested in 2 mL PBS containing 20 mM HEPES, 5% fetal bovine serum (FBS), 60 U/mL DNase I (Sigma #11284932001), and 450 U/mL collagenase type I (Sigma #SCR103) at 37°C for 45 min with gentle agitation every 15 min. Cell suspensions were centrifuged at 350 × *g* for 5 min, lysed with 1 mL ACK buffer for 5 min, and neutralized with FACS buffer. After filtration through a 40 μm strainer, cells were centrifuged again at 350 × *g* for 5 min, and resuspended in ice-cold FACS buffer.

#### 2.6.4 | Aorta

To make a single-cell suspension from the aorta, mice were perfused by cardiac puncture with 10 mL PBS. Mouse aorta (from ascending thoracic aorta to abdominal aorta) was collected and washed twice with PBS to remove blood cells in the vessel. Mouse aortas were microdissected into 2 mm length and digested in 2 mL PBS containing 20 mM HEPES/5% FBS with 2 mL/aorta of 125 U/mL collagenase type XI (MilliporeSigma, 9001-12-1), 60 U/mL hyaluronidase type I-s (MilliporeSigma, 37326-33-3), 60 U/mL DNAse1 (MilliporeSigma, 11284932001), and 450 U/mL collagenase type I (MilliporeSigma, SCR103) at 37°C for 45 min. The incubation mixture was shaken every 15 min. The digested aorta was passed through a 70 μm strainer, and the strainer was rinsed with FACS buffer. Cells were centrifuged at 350 g for 5 min at 4°C, and the aortic cells were resuspended in ice-cold FACS buffer.

Cell suspension was stained with fluorescence conjugated antibodies (Table 1) and analyzed using an LSRII flow cytometer (BD Biosciences) at the Temple University FACS Core Facility. Flow cytometry data were analyzed using FlowJo v10 software with the Proliferation Wizard platform (https://www.flowjo.com/flowjo10/overview).

#### 2.6.5 | Immune Fluorescence Staining

Aortic sinus sections were fixed with 4% paraformaldehyde and permeabilized with 0.1% Triton X-100. Following blocking with 5% BSA buffer, sections were incubated overnight at 4°C with primary antibodies against CD155 (Rabbit-anti-Mouse, R&D system, MAB6909), CD68 (Rat-anti-mouse, Invitrogen, 14-0681-82), and CD206 (Goat-anti-Mouse R&D system, AF2535). After washing with PBS, sections were incubated with Donkey-anti-Rabbit (AF647, Invitrogen, A31573), Donkey-anti-Rat (AF568, Invitrogen, A78946), and Donkey-anti-Goat (AF488, Invitrogen, A11055) secondary antibodies for 1 h at room temperature in the dark. Nuclei were counterstained with VECTASHIELD Vibrance Antifade Mounting Medium with DAPI (Vector Laboratories, H-1800–10). Sections were imaged using a Leica SP8 confocal laser-scanning microscope. Images were acquired using identical imaging settings for comparable experimental groups.

### 2.7 | Statistical Analysis

Data are expressed as mean ± standard error of the mean (SEM). Statistical comparisons between two groups were performed using a two-tailed Student's *T*-test. For comparisons among three or more groups, one-way ANOVA followed by Tukey's test was applied. Flow cytometry data represents 2–3 independent biological replicates. A *p* value < 0.05 was considered statistically significant.

## 3 | Results

### 3.1 | Atherosclerotic Tregs Exhibit Sustained Immunosuppression Signaling and Increased Expression of Inhibitory Checkpoint Receptors

Pathway analysis of RNA-seq data revealed that CD4^+^Foxp3^+^ Tregs from ApoE^−/−^ mice displayed activated and sustained immunosuppression signaling compared to WT Tregs (Figure 2A,B). Given that inhibitory ICP receptors promote Treg immunosuppression and limit T cell activation and effector function, we next examined their expression profiles of ICP receptors. The results showed that most of the inhibitory ICP receptors, which enhance suppressive function, but also the majority of stimulatory ICP receptors, which counteract suppressive function, were upregulated in atherosclerotic Tregs (Figure 2C). Functional annotation further identified APC differentiation as one of the three significant downstream effects enriched in ApoE^−/−^ Tregs relative to WT controls (Figure 2D). These results suggest that a dynamic equilibrium between coinhibitory and costimulatory signaling may shape Treg-mediated immune regulation during atherosclerosis progression. To further explore this, we used the IPA platform to identify datasets exhibiting significant activation or inhibition of APC differentiation (|*z* score| > 2) in atherosclerosis studies. In addition to our Treg dataset, 14 independent comparisons from seven other datasets were included for further analysis (Figure 2E, Table S2).

### 3.2 | Dynamic Expression of Dual-Function Immune Checkpoint Ligand CD155 in Atherosclerotic Aorta Under Chow and High-Fat Diet Conditions

Building on our previous analysis of 28 ICP receptors and ligands across 32 human and mouse tissues, tumors, and polarized macrophages and endothelial cells (ECs) [4], we expanded our dataset to include 58 reported receptors–ligands pairs [8] (Table S1) and investigated their expressions in atherosclerotic cardiovascular disease (ASCVD) [15, 42, 43, 45]. After analysis, we selected stimulatory and inhibitory ICP pairs that showed significant changes during atherosclerotic development. Overall, more ICPs were upregulated than downregulated in both human and mouse arteries (Figure 3**, datasets 4**–**15**). In contrast, at the cellular level (Figure 3, datasets 1–3), the numbers of increased and decreased ICPs were comparable. Consistent with previous findings, CD28 and CD80 were significantly upregulated in human atherosclerotic arteries and in advanced-stage atherosclerotic mouse aortas, indicating dominant costimulatory signaling during atherogenesis. The dual-function ligand CD155 (capable of binding to immunosuppressive IC receptors TIGIT, CD96 and costimulatory IC receptor CD226) [46] was significantly reduced in human plaque versus nonplaque areas of carotid and peripheral arteries. Similarly, in atherosclerotic mice, CD155 expression was modest but significantly declined under HFD conditions, while exhibiting dynamic changes during chow diet feeding compared to WT controls. Among inhibitory receptors, TIGIT displayed the highest log_2_FC (0.81) in ApoE^−/−^ Tregs, though its expression was not significantly altered in bulk human or mouse aortic transcriptomic data—likely due to signal dilution, as TIGIT expression is largely restricted to Tregs [47]. Conversely, CD96, another inhibitory IC receptor for CD155, was downregulated in ApoE^−/−^ Tregs but upregulated in human and in mouse arteries under HFD condition, along with the costimulatory IC receptor CD226. Of note, the CD274/PD-1 pair was induced in the later stages of atherosclerotic mouse model, aligned with previous reports [48] but did not show consistent changes in human arteries.

Other relevant changes included upregulation of IC receptor HAVCR2 (TIM3) in both species, although its ligands showed minimal alterations. A stimulatory pair TNFRSF9/TNFSF9 also exhibited increased receptor expression without corresponding ligand induction. LAG3 was robustly induced in ApoE^−/−^ Tregs and upregulated in human plaques, together with its ligand LGALS3, though only LGALS3 was increased in the mouse model. The CD48/CD2/CD244 axis demonstrated species-specific expression differences, with all three members upregulated in mouse arteries, whereas only CD2 and CD244 were increased in human samples. Collectively, these findings demonstrate species- and context-specific expression patterns of ICPs in atherosclerosis, emphasizing that both stimulatory and inhibitory signaling pathways contribute to disease pathogenesis and progression.

### 3.3 | Single-Cell RNA-Sequencing Reveals Cell-Type-Specific ICP Expression in Atherosclerosis

The inconsistent expression changes in ICPs among Tregs, aortic foam cells, and arterial tissues underscore the cell-type specificity of ICP expression and highlight the importance of cell-specific regulation of the ICP axis during ASCVD. To gain deeper insights into cell-type-specific ICP expression in atherosclerosis, particularly for those that have not been extensively studied, we performed single-cell RNA sequencing (scRNA-seq) analysis of three published datasets from ApoE^−/−^ and low-density lipoprotein receptor (Ldlr)^−/−^ *mouse aortas*. Heatmaps of ICP expressions were generated using log_2_FC values (with significance) of each cell cluster compared to other cells (Figure 4). Because only CD45^+^ immune cells from mouse aorta were sequenced, a higher resolution analysis of ICP expression in aortic immune cells was revealed than that in the literature. The cell types enriched for CD86 differed between ApoE^−/−^ and Ldlr^−/−^ mice under HFD conditions. In ApoE^−/−^ mice, B cells exhibited higher CD86 expression, along with monocyte-derived dendritic cells DCs/DCs and macrophages compared to other cell types. In contrast, in Ldlr^−/−^ mice, CD86 enrichment was observed only in monocyte-derived DCs/DCs and macrophages. CD226, a stimulatory receptor for CD155, was enriched in mixed T cell clusters, whereas the expression of CD96, an inhibitory receptor, was more specific to CXCR6^+^ T cells. Interestingly, CD155 expression appeared in a mixed T cell cluster in Ldlr^−/−^ mice, although this cluster differed from the one enriched for CD226. These findings broaden our understanding of ICP receptor–ligand interactions, suggesting they may extend beyond the classical APC–T cell interaction and warrant further investigation. It is worth noting that no TIGIT enrichment was observed in either ApoE^−/−^ or Ldlr^−/−^ mice, likely due to the lack of defined Treg clusters in these datasets.

Havcr2 (TIM3), which was induced during atherosclerosis progression, was enriched in monocytes. Conversely, Lgals3 was enriched in macrophage subsets in ApoE^−/−^ mice, corresponding to resident-like macrophages in Ldlr^−/−^ aorta [44], whereas Lgals3 was enriched in TREM2^high^ macrophages in Ldlr^−/−^ datasets. In 12-week HFD-fed ApoE^−/−^ mouse aorta, CD48 was enriched in CD8^+^ T cells and macrophages, whereas CD2 was enriched in T cells and natural killer (NK) cells. In 20-week HFD-fed Ldlr^−/−^ mouse aorta, CD48 was specifically enriched in TREM2^high^ macrophages, which have specialized functions in lipid metabolism and present a gene expression signature reminiscent of osteoclasts, suggesting a role in atherosclerotic lesion calcification [44]. Itgb1, decreased in both human and mouse atherosclerotic arteries, was enriched in T cells, particularly CD8^+^ T cells, and NK cells, whereas Lair1, consistently increased in human and mouse arteries, was enriched in macrophages, especially inflammatory macrophages. These data identify novel ICP–cell associations and potential therapeutic targets in atherosclerosis.

### 3.4 | CD155 Is Upregulated in Aortic CD45+ Immune Cells Despite Its Decrease in Whole Aorta, Accompanied by Increased TIGIT Expression in Aortic Tregs of ApoE−/− Mice

Immune checkpoint receptors critically regulate CD4^+^Foxp3^+^ Tregs [4, 49, 50]. Our previous study identified new subsets of immunosuppression-sustained CD4^+^Foxp3^+^ Tregs in ApoE^−/−^ mice, driven by the Treg-generated anti-inflammatory and immunosuppressive cytokine interleukin-35 (IL-35) [15]. RNA-seq data confirmed that both inhibitory and stimulatory ICPs were upregulated in splenic Tregs from HFD-fed ApoE^−/−^ mice compared to WT controls [15]. However, we observed higher expression of inhibitory ICP receptors, including TIGIT and PD-1, in a Treg subset associated with sustained immunosuppressive function compared with that of Tregs^TIGITlow^ and Tregs^PD−1low^ cells. This upregulation is associated with activated phenotypes and increased expression of migratory molecules in Tregs. Among the upregulated inhibitory ICP receptors in Tregs, TIGIT demonstrated higher induction scales (log_2_FC). Since Tregs are a small cell population in the aorta, it is technically challenging to isolate sufficient Treg numbers for RNA-seq-based transcriptomic analyses. Limited expression data for ICP receptors and ligands on Treg populations were identified in single cell RNA-seq datasets from both human and mouse aorta studies, demonstrating their pathophysiological relevance in aortic pathologies.

To investigate the expression and potential roles of TIGIT and its ligand CD155 in atherosclerosis development, we performed qRT-PCR on aortas from 12-week HFD-fed ApoE^−/−^ and WT mice, consistent with the mouse model and time course used in our RNA-seq study [15]. CD155 was significantly decreased in ApoE^−/−^ aortas (Figure 5A), whereas TIGIT transcripts were below detection. Flow cytometry analysis of CD45^+^ and CD45^−^ fractions revealed that CD155 was significantly upregulated in CD45^+^ leukocytes but not in CD45^−^ vascular cells (Figure 5B). These findings align with various array transcriptomic datasets, indicating that leukocytes, including monocyte-derived macrophages and circulating proangiogenic cells that differentiate into APCs, exhibit CD155 induction in response to pro-inflammatory stimuli. However, in our newly identified innate immune cells [51–53], aortic ECs, exposed to hyperlipidemia or in aortic nonplaque areas analyzed using laser capture microdissection, CD155 levels were decreased (Figure 5C). Further comparison of CD155 expressions in CD3^+^ and CD3^−^ cell fractions within aortic CD45^+^ leukocytes revealed that CD155 expression increases in ApoE^−/−^ mice compared to WT controls were localized to non-T cells (Figure 5D). Next, we examined TIGIT expressions in aortic T cells. Like its expression in splenic Tregs, TIGIT was significantly induced in aortic Foxp3^+^ Tregs, but not in Foxp3^−^ T cells from atherosclerotic ApoE^−/−^ mice compared to that of WT controls (Figure 5E). Together, these data suggest that the TIGIT-CD155 inhibitory axis plays an active role in interactions between aortic Tregs and APCs under hyperlipidemia conditions. This interaction may enhance the immunosuppressive functions of Tregs [2, 10, 11, 15, 54–58], thereby impeding the inflammatory progression of atherosclerosis [15, 39] (Figure 5F).

### 3.5 | Flow Cytometry Analysis Identified That CD155 Expresses in Parallel With CD206 in CD11b^+^CD11c^+^ and CD11b^−^CD11c^+^ Cells

To further identify potential cellular subsets for studying the TIGIT-CD155 axis in CVDs, CD45^+^ immune cells were profiled by flow cytometry from blood, lung, and kidney. Among T cells (CD45^+^CD3^+^), the percentages of TIGIT^+^ cells were significantly higher in CD4^+^Foxp3^+^ Tregs compared to CD4^+^Foxp3^−^ effector T cells (Teffs) and CD8^+^ cells in both blood and lung (Figure 6A). In the kidney, TIGIT expression levels were comparable among Tregs, CD4^+^ Teffs, and CD8^+^ T cells, with some variations likely due to the limited number of CD45^+^ cells collected from mouse kidneys. No significant differences in TIGIT^+^ T cells from kidneys were observed between ApoE^−/−^ and WT control mice under chow diet feeding conditions.

Among non-T (CD45^+^CD3^−^) cells, four distinct populations were identified based on the expression of CD11b (myeloid marker) and CD11c (DC marker) in blood, lung, and kidney. As discussed by previous study and our flow cytometry costaining (data not shown), CD11b^+^CD11c^−^ (CD11b single positive, CD11b SP) cells predominantly comprise monocytes, whereas CD11b^+^CD11c^+^ cells (double positive, DP) are likely originating from monocytes and representing a heterogeneous population of macrophages and DCs. CD11b^−^CD11c^+^ (CD11c SP) cells primarily comprise DCs [40, 41]. When comparing the frequencies of these populations between ApoE^−/−^ and WT mice, CD11b SP cells were highly increased in blood, DP cells were increased only in the lung, and CD11c SP cells were increased in all three tissues in ApoE^−/−^ mice (Figure 6B). Next, we profiled CD155 expressions in these populations (Figure 6C). CD155 expression was higher in DP cells in blood and kidney and in both DP and CD11c SP cells in the lung. Combining the increased frequency of CD11c SP cells in ApoE^−/−^ mice with their mean CD155 expression levels, CD11cSP DCs emerged as major potential targets of the TIGIT/CD155 axis in the lung and kidney. Of note, CD155 expression was downregulated in DP cells in the lung and kidney of ApoE^−/−^ mice compared to WT, which contrasts with previous reports of similar cells in atherosclerotic aortas [42]. These discrepancies may reflect tissue specificity or chow diet feeding conditions, which need further confirmation. Given that CD155 enrichment was also observed in mixed T cells from atherosclerotic aortas in Ldlr^−/−^ scRNA-seq data, we further examined CD155 expression in CD45^+^CD3^+^ T cells and non-T cells across the three tissues (Figure 6D,E). The mean CD155 levels were lower in T cells compared to DP or CD11c SP cells, and all populations exhibited downregulation of CD155 in ApoE^−/−^ mice compared to WT. These findings suggest that the TIGIT/CD155 axis may play a more prominent role in Treg-DC interactions during the early stages of hyperlipidemia.

To identify CD155 expression in three functionally distinct macrophage-DC subsets [43], we analyzed three surface markers: CX3CR1, CD103, and CD206 expression. CX3CR1 is a chemokine receptor in the CX3CL1/CX3CR1 axis that mediates the migration and recruitment of immune cells during inflammation, as reported by us and other researchers [59, 60]. CD103 is an integrin protein expressed on immune-enhancing DCs [61] and immunosuppressive Tregs [62], playing an important role in maintaining immune homeostasis. CD206 is a C-type lectin expressed on anti-inflammatory M2-like macrophages [63] and immature DCs [64]. Our results showed that: (1) CX3CR1, CD103, and CD206 were barely expressed in CD11b SP cells across all tissues; (2) DP and CD11c SP cells expressed varying levels of these markers, with DP cells in the lung exhibiting higher levels compared to those in blood and kidney; and (3) CD11c SP cells in the lung showed higher levels of CD206, whereas those in the kidney expressed higher levels of CX3CR1 and CD103 (Figure S1). The results indicate that DP DCs and CD11c^+^ SP DCs exhibit greater heterogeneity across tissues than other APC populations, highlighting the need for further comparison of CD155 expressions in these subsets.

To determine whether CD155 expression was differed within DP and CD11c^+^ SP subsets based on coexpression of CX3CR1, CD103, or CD206, these cells were categorized into CX3CR1^+^, CX3CR1^−^, CD103^+^, CD103^−^, CD206^+^, and CD206^−^ subsets. As judged by the absolute value of CD155 mean fluorescence intensity and the expression levels of the functional markers in Figure 6F, the results showed that: (1) in blood DP cells, CD155 expression was higher in the CX3CR1^−^ subset than in the CX3CR1^+^ subset. However, in blood CD11c^+^ SP cells, CD155 expression was higher in the CX3CR1^+^ subset than in the CX3CR1^−^ subset, suggesting a cooperative role of CD11c and CX3CR1 in upregulating CD155 in blood. (2) In lung and kidney DP cells, CD155 expression was higher in the CD103^+^ subset than in the CD103-subset. However, in lung CD11c SP cells, CD155 expression was lower in the CD103^+^ subset compared to the CD103^−^ subset, indicating that CD11b and CD103 may work together to upregulate CD155 in the lung. (3) CD155 expression was significantly higher (2-fold or more) in the CD206^+^ subset compared to the CD206^−^ subsets across both DP and CD11c SP cells in all tissues. These results suggest that CD155 expression is positively correlated with CD206 levels in CD11b^+^CD11c^+^ and CD11c SP cells. Importantly, this finding was further validated in the aortas of ApoE^−/−^ mice following 12 weeks of Western diet feeding (Figure 6G). Furthermore, immunofluorescence staining of the aortic sinus revealed a patchy CD155 staining pattern, with CD155 detected in a subset of CD68^+^CD206^+^ cells surrounding the aortic sinus lesion, whereas additional punctate CD155 signals were observed within the plaque, particularly in regions with limited nuclear staining and without clear colocalization with CD68 (Figure 6H). Together, these findings support a potential association between CD206^+^ macrophage subsets and CD155 expression, and suggest a potential role for CD206^+^ subsets within CD11b^+^CD11c^+^ and CD11ĉSP cells in TIGIT–CD155-mediated immune regulation during hyperlipidemia.

## 4 | Discussion

Recent progress has highlighted the significant role of ICP receptors and ligands in regulating the pathogenesis of CVD [4, 8, 65]. Our previous work also identified that atherosclerosis Tregs display sustained immunosuppression, including elevated expression of inhibitory ICP receptors [66]. To gain a comprehensive understanding of ICP expression in atherosclerosis, we initially screened 58 pairs of ICP receptors and ligands across human and mouse atherosclerotic vessel and cell samples, leading to several key findings: (1) More ICPs are upregulated than downregulated in both human and mouse arteries, whereas at the cellular level, for examples, in aortic foam cells, the numbers of increased and decreased ICPs in each category are comparable. (2) In atherosclerotic Tregs, the inhibitory ICP receptor TIGIT is the most upregulated compared to WT Tregs, whereas its ligand, the dual-function molecule CD155, shows dynamic fluctuations during chow diet feeding but is consistently downregulated under HFD conditions in both mice and humans. (3) scRNA-seq revealed cell-type-specific expression of ICPs in atherosclerosis and the expression of CD155 broadened the understanding of ICP receptor–ligand interactions beyond the classical APC -T cell paradigm; (4) The TIGIT-CD155 axis is upregulated in aortic CD45^+^ immune cells in ApoE^−/−^ mice, and the mannose receptor CD206 expresses in parallel with CD155 in CD11b^+^CD11c^+^ monocytic macrophages and DCs, as well as in CD11b^−^CD11c^+^ nonmonocytic DCs. Our findings provide a comprehensive overview of the differential expression of 58 pairs of ICP receptors and ligands in atherosclerosis. They highlight the correlation between CD155 expression and CD206 in the initiation of atherosclerosis and show that the CD155–TIGIT axis is predominant in atherosclerosis compared to other CD155 interaction pairs, such as CD155-CD96 and CD155-CD226.

Early studies have provided compelling evidence that ICP axes are involved in the progression of atherosclerosis [67]. It is noted that regulation of signaling strength need not necessarily occurs through changes in both ligand and receptor expression level [68]; however, understanding which cell types play major roles in the ICP axis is of vital importance for determining the pathological outcome. Building on our previous RNA-sequencing results on Tregs, we selected CD155 as the target for further study. Combining microarray analysis, scRNA-seq, and flow cytometry validation, we found that CD155 expression is increased in the CD45^+^ immune cell compartment of the mouse aorta but decreased in the adventitia—both within and outside the plaque—as well as in ECs, despite an overall reduction of CD155 expression in atherosclerotic vessels. Therefore, omics data analysis helps us uncover changes in small cell populations that may be masked by the signals from larger compartments.

CD155 is an adhesion molecule in the Nectin-like family that is highly upregulated in multiple cancer types and is associated with poor patient outcomes [69]. CD155 signals to immune cells through interactions with the costimulatory ICP receptor CD226 (DNAM-1) and two inhibitory ICP receptors, TIGIT and CD96, on T cells and NK cells. During tumor progression, inhibitory receptors for CD155 are upregulated on effector T cells, impairing their cytotoxic capacity [70]. Meanwhile, CD155 promotes the degradation of CD226 on tumor-infiltrating CD8^+^ T cells, facilitating immune evasion and resistance to cancer immunotherapy [71]. The integration of signals from CD155's cognate receptors modify the activity of tumor-infiltrating lymphocytes in a context-dependent manner, making CD155 an attractive target for immuno-oncology [69]. Our findings reveal that CD155 is expressed in parallel with the anti-inflammatory macrophage marker CD206, highlighting CD206^+^ macrophages and DCs as the primary cell populations involved in the CD155–TIGIT/CD226/CD96 axis in the regulation of atherosclerosis. Of note, CD206 may contribute to the anti-inflammatory phenotype through both intragenic miR-511–3p regulation [72] and ligand-induced receptor signaling [73]. However, whether these mechanisms regulate or cooperate with the CD155–TIGIT/CD226/CD96 axis in atherosclerosis remains unexplored.

TIGIT, a high-affinity receptor for CD155, exerts diverse immunosuppressive effects, including inhibition of NK cell function, suppression of DC maturation, promotion of M2 macrophage polarization, and Tregs differentiation [74]. Multiple coinhibitory checkpoints are significantly induced in Tregs from 12-weeks HFD-fed ApoE^−/−^ mice compared to those from WT control, with TIGIT induction ranked the highest [15]. Using flow cytometry, we identified that TIGIT expression is increased in atherosclerotic Tregs and that its expression in Tregs is higher than other T cell subsets. These results also explain why TIGIT expression is difficult to detect in tissue transcriptomic arrays or even in scRNA-seq, as it is primarily enriched in a relatively rare population. Additionally, TIGIT competes with CD226, CD96 for CD155 binding with higher affinity [75], suggesting that the CD155–TIGIT axis is activated in immune cells under atherosclerotic conditions and may play a protective role against lesion progression.

Macrophages, DCs, and B cells are traditionally regarded as classical APCs in the immune system. However, recent research has identified additional cell types—including mast cells, basophils [76], eosinophils, neutrophils, innate lymphoid cell 3, lymph node stromal cells, and epithelial cells—as potential APCs capable of presenting antigens on MHC class II molecules to CD4^+^ T cells [77]. We have also reported transcriptomic evidence suggesting that ECs [51, 78], VSMCs [79], and potential APC-like Tregs [2] may function as APCs.

The current working model posits that ICP receptors are expressed on T cells and ICP ligands on APCs. However, our data demonstrates that ICP receptors are expressed on non-T cells in aortic diseases, and ICP ligands, such as CD155, can be expressed on T cells. For instance, CD155 at intercellular junctions in vascular ECs regulates monocyte migration by binding to CD226 on monocytes [80]. This expands the understanding of ICP receptors–ligands interaction beyond classical APC-T cell interaction, showing their involvement in diverse cell types in CVD. Immune heterogeneity indicates that ICPs are involved not only in regulating immune cells but also nonclassical immune cells in CVDs [51, 52].

Our findings propose a new working model: (1) Sustained immunosuppression was activated in ApoE^−/−^ Tregs, highlighting the significance of the ICP axis in regulating atherosclerosis progression; (2) Omics data display the overview of ICP receptors and ligands' transcriptomic response in atherosclerosis across tissues, cell types, and disease stages; (3) We identified potential ICP targets, such as CD155 (as dual-function ligands), Lgals3 and Itgb1 (as inhibitory ligands), Tnfrsf9 and CD48 (as stimulatory ligands), and Havcr2 and Lair1 (as inhibitory receptors) are significant sensors of microenvironmental changes associated with atherosclerosis by comparing human and mouse disease models. (4) Experimental verification addressed the upregulation of CD155-TIGIT in the recruited immune cells in atherosclerosis plaques and revealed that CD155 expressions are higher in CD206^+^ subset than CD206^−^ subset in CD11b^+^CD11c^+^ DP and CD11^+^ SP cells regardless of tissue type; (5) The CD206^+^ MØs/DCs–CD155–TIGIT^+^ Treg axis is a potential ICP pathway that protects against atherosclerosis progression. These results highlight the previously unrecognized innate immune functions of ICP ligands in atherosclerosis, extending beyond their established roles in regulating T cells and Tregs, as reported in cancer therapy [81]. These findings reveal previously unrecognized immune functions of ICP ligands in atherosclerosis, extending their role beyond T cell and Treg regulation, as traditionally described in cancer immunotherapy, to include direct involvement in modulating innate immune responses within atherosclerotic lesions.

A limitation of the present study is that, although we identified differential expression and associations of ICP ligand–receptor pairs, particularly CD155/TIGIT, in atherosclerosis, we did not perform gain- or loss-of-function experiments to establish their causal roles in disease progression or immune-cell function. Therefore, our findings should be interpreted primarily as descriptive and associative rather than as evidence of direct mechanistic relationships. Importantly, our findings do not exclude potential immunostimulatory effects of CD155 through other receptors, such as CD226. The functional consequences of CD155 signaling may depend on receptor expression and cellular context. Further studies using appropriate genetic or pharmacological approaches are needed to determine the respective contributions of CD155/TIGIT, CD155/CD226, and CD155/CD96 signaling to atherosclerosis and their therapeutic potential. Finally, most mouse experiments were performed using male mice, which may limit the generalizability of our findings to female mice. Studies including both sexes will be important to determine whether these ICP associations are sex-dependent.

## Supplementary Material

Table S1

Table S2

Figure S1

Additional supporting information can be found online in the Supporting Information section.

**Figure S1:** Three functional marker CX3CR1 CD103 and CD206 are comparably highly expressed in CD11b^+^CD11c^+^ (monocytic macrophages and DCs) and CD11b^−^CD11c^+^ (nonmonocytic DCs) cell subsets than in CD11b^+^CD11c^−^ monocytes and double negative cells in blood, lung and kidney. **Table S1:** Immune checkpoint couples (58 pairs). **Table S2:** Dataset details included in this study.

## Figures and Tables

**FIGURE 1 |:**
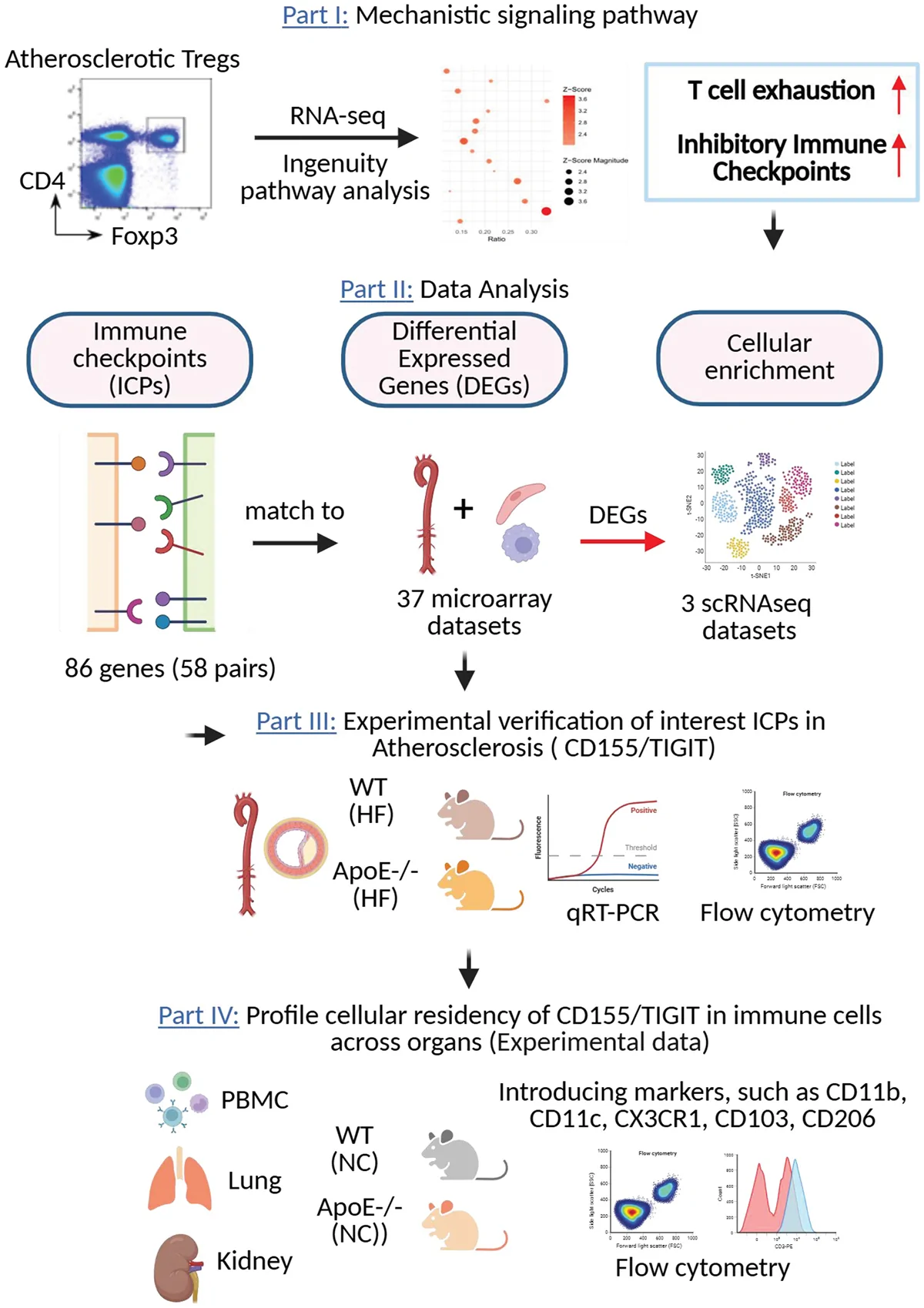
A workflow chart and strategies of this work.

**FIGURE 2 |:**
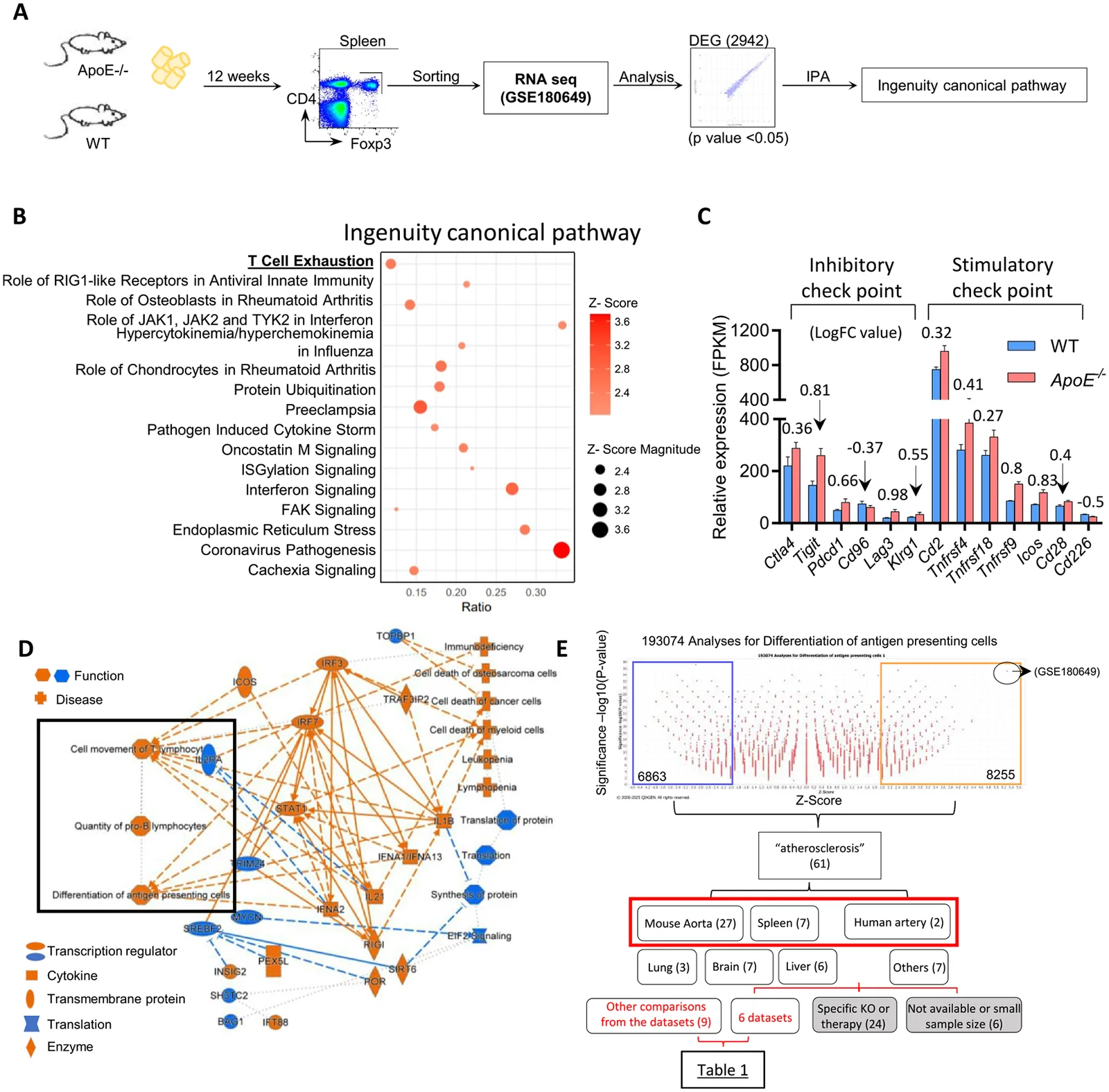
Atherosclerotic Tregs exhibit sustained immunosuppression signaling and increased expression of inhibitory checkpoint receptors. (A) RNA sequencing analysis was performed on splenic CD4+Foxp3+ Tregs isolated from Ape^−/−^ mice (*n* = 4) and wild-type (WT) controls (*n* = 3) fed a high-fat diet for 12 weeks. A total of 2942 differentially expressed genes were identified and subjected to ingenuity pathway analysis (IPA). (B) Bubble plot showing significantly activated signaling pathways filtered by −log10 (*p*-value) > 1.3 and *Z*-score > 2. (C) Bar chart depicting differentially expressed inhibitory and stimulatory immune checkpoint genes in Ape^−/−^ Tregs relative to WT controls. Expression values are represented as fragments per kilobase of transcript per million mapped reads (FPKM), with the log2 fold change indicated above each bar. (D) IPA-generated graphical summary illustrating predicted diseases and functions (highlighted in black rectangle) associated with the Treg transcriptome, including “differentiation of antigen-presenting cells (APC).” (E) A total of 193,074 datasets were retrieved from the IPA database annotated to “differentiation of APC.” Among these, our RNA-seq dataset exhibited the highest *Z*-Score and significance. Further filtering by |*Z*-score| > 2 and the key word “atherosclerosis” identified 61 atherosclerosis-related datasets. The flowchart outlines the selection process leading to 15 datasets chosen for downstream analysis.

**FIGURE 3 |:**
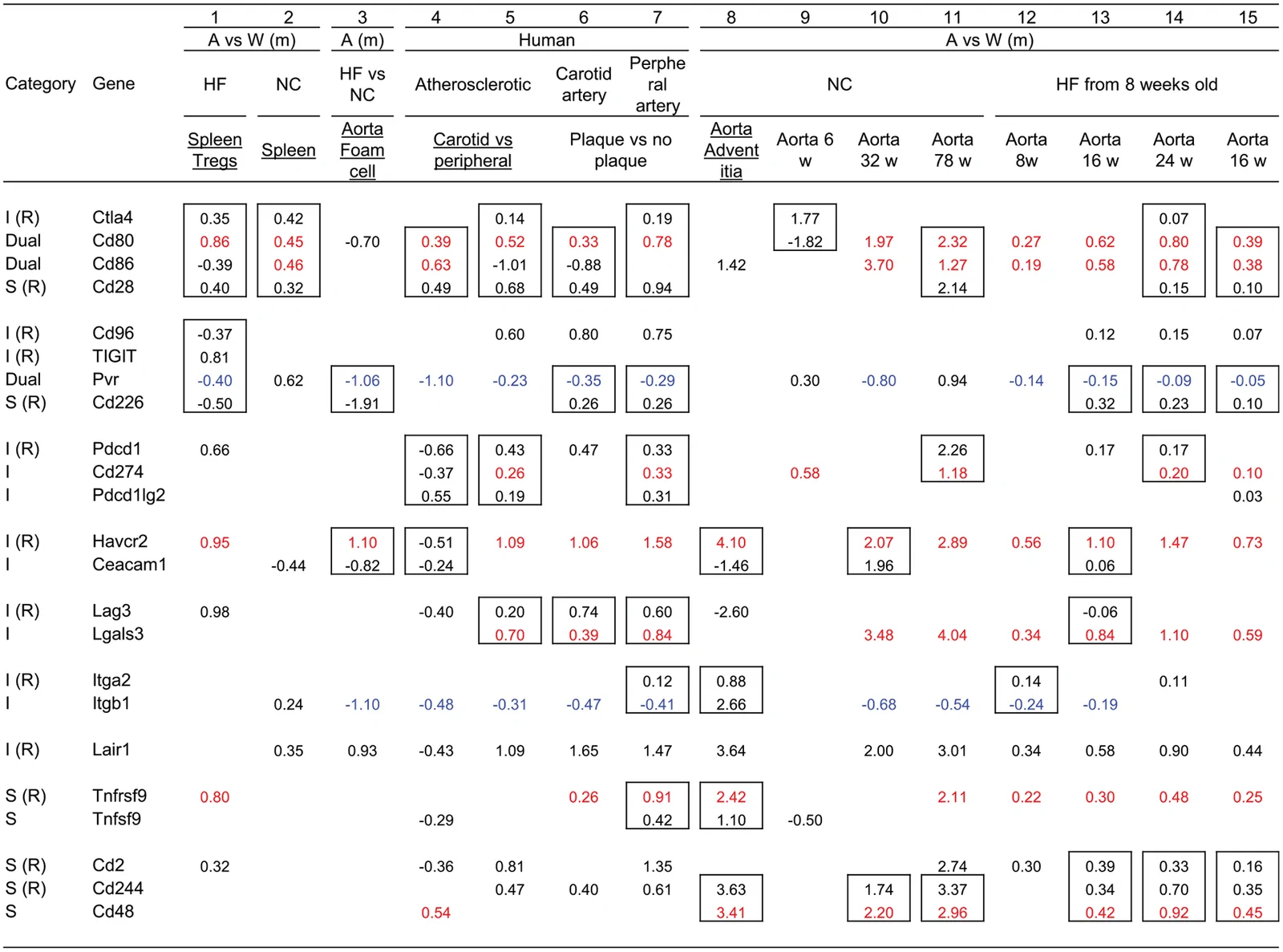
The dual-function ICP ligand CD155 exhibits dynamic regulation during APC differentiation and decreases under high-fat diet conditions in the atherosclerotic aorta. Selected stimulatory and inhibitory ICP ligand–receptor pairs showing differential regulation during APC differentiation and within the atherosclerotic aorta. Ligands exhibiting increased or decreased expression are highlighted in red and blue, respectively. A: atherosclerosis; APC: antigen-presenting cell; HF: high-fat diet; I: inhibitory; ICP: immune checkpoint; NC: normal chow diet; R: receptor; S: stimulatory; vs.: versus; w: weeks; W: wild-type.

**FIGURE 4 |:**
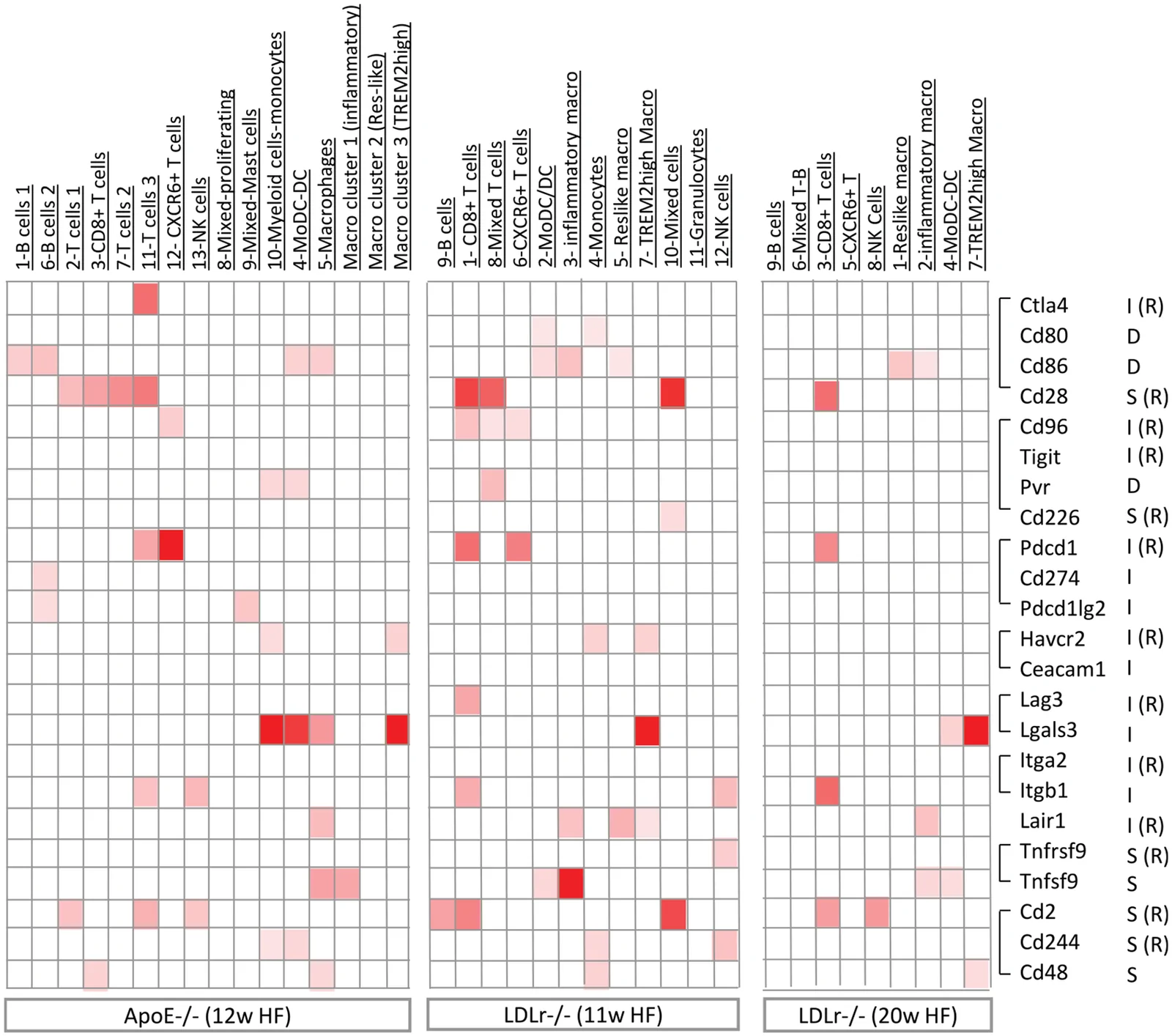
Single-cell RNA sequencing analysis reveals cell type-specific expression of immune checkpoint (ICP) genes in aortic cells from atherogenic mouse models. Heatmap analysis of three single-cell RNA-seq datasets illustrating the expression patterns of selected ICP genes identified in Figure 2B, stratified by cell type. Log_2_ fold-change (Log_2_FC) values represent the difference in the percentage of cells expressing a given gene within each cluster compared to those outside the cluster. Notably, CD155 (PVR) expression was enriched in myeloid populations, including monocytes and monocyte-derived dendritic cells (mono-DCs), in the aortas of ApoE^−/−^ mice (12 weeks on a high-fat diet, HFD), and in mixed T-cell populations in the aortas of LDLR^−/−^ mice (11 weeks on HFD). In contrast, CD274 (PD-L1) expression was predominantly enriched in B cells from ApoE^−/−^ aortas. No TIGIT expression data were available in the analyzed datasets.

**FIGURE 5 |:**
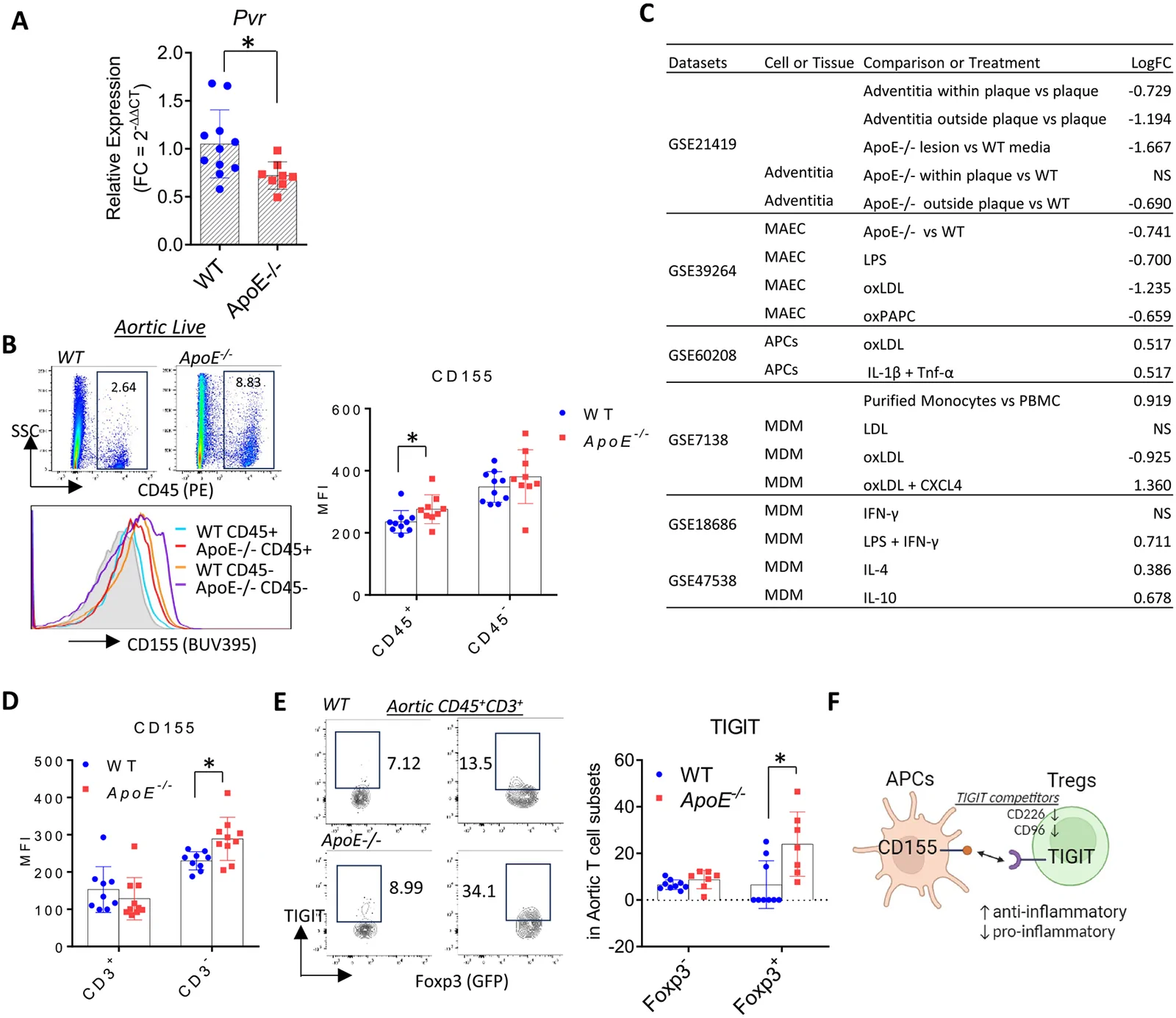
CD155 is selectively upregulated in ApoE^−/−^ aortic CD45+ immune cells but decreased in whole aorta compared to WT controls; and CD155 binding receptor TIGIT protein expression was also specifically increased in ApoE^−/−^ aortic CD4+Foxp3+ regulatory T cells (Tregs) compared to WT controls. Twelve-week high-fat diet (HFD)-fed ApoE^−/−^ and WT mice (*n* > 8 per group) were analyzed. (A) CD155 mRNA expression in whole aorta was quantified by real-time PCR. (B) CD155 protein expression (mean fluorescence intensity, MFI) was measured in aortic CD45+ immune cells and CD45-nonimmune cells (at the single-cell levels) using flow cytometry (FACS). CD155 expression was significantly elevated in CD45^+^ immune cells from ApoE^−/−^ mice compared with WT controls. (C) Differential CD155 expression across multiple transcriptomic datasets, including comparisons between different areas of the mouse atherosclerotic aorta (GSE21419), mouse aortic endothelial cells (MAECs) (GSE39264), antigen-presenting cells (APCs) (GSE60208), and monocyte-derived macrophages (MDM) (GSE7138, GSE18686, GSE47538). (D) CD155 expression in aortic CD3+ T cells and CD3-non-T cell subsets in CD45+ cells in ApoE^−/−^ mice compared with WT controls, showing significantly increased CD155 expression in the non-T cell compartment. (E) TIGIT protein expression was markedly increased in aortic Foxp3+ Tregs from ApoE^−/−^. As a control, there was no change in the expression of TIGIT in aortic CD3+ Foxp3− T cells between ApoE^−/−^ and WT mice. (F) Schematic representation of the proposed mechanism by which the CD155/TIGIT immune checkpoint pathway contributes to vascular inflammation in atherosclerosis. WT, wild-type.

**FIGURE 6 |:**
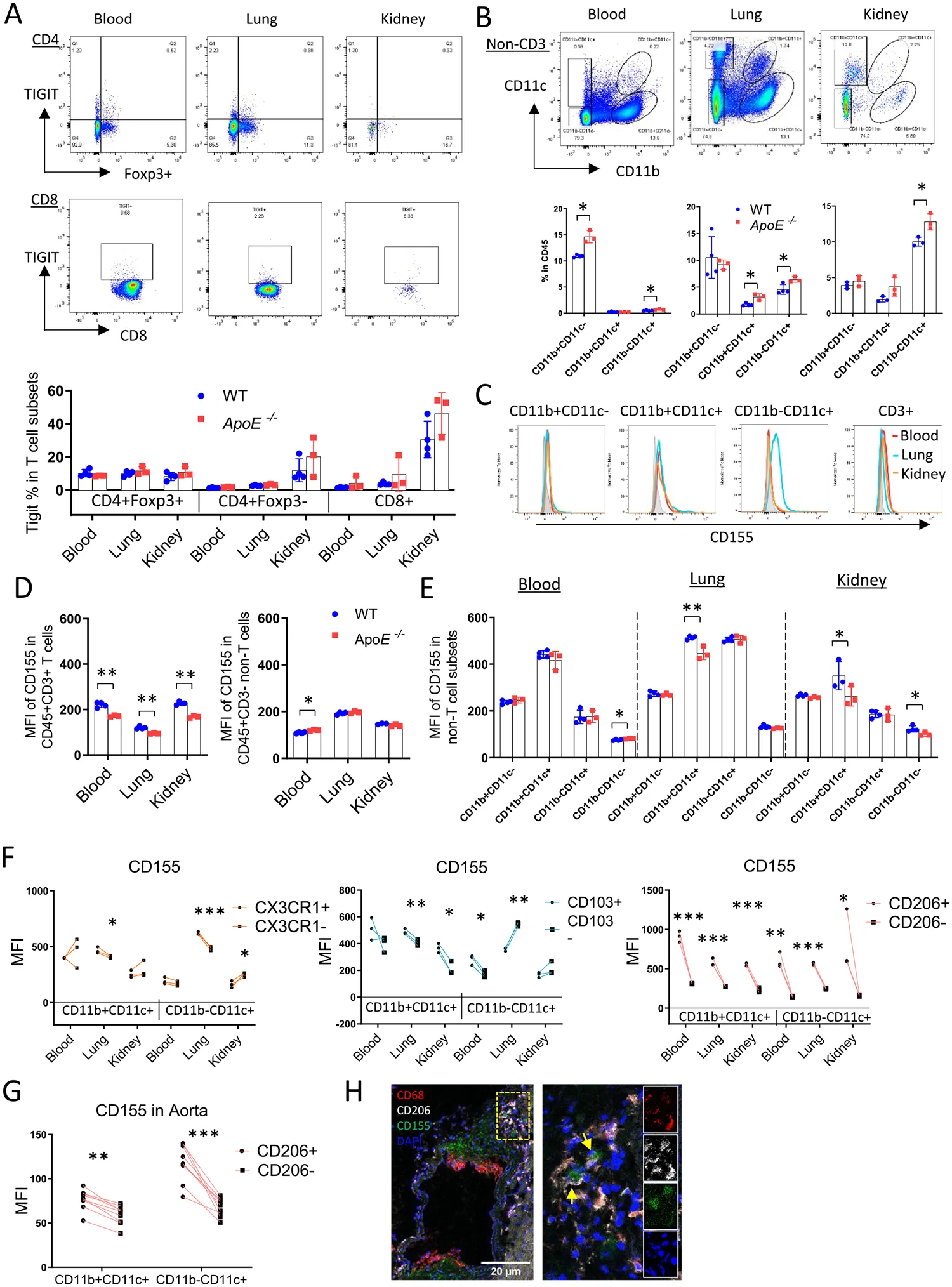
Flow cytometric analysis reveals that CD206 expression is associated with upregulation of CD155 in CD11b^+^CD11c^+^ cells and CD11b^−^CD11c^+^ cells across multiple tissues. 12-wks-normal chow diet (CD)-fed ApoE^−/−^ (*n* = 3 per group) and WT mice (*n* = 4 per group) were used for analysis. (A) Representative flow cytometry plots showing the percentages of TIGIT^+^ cells within CD4^+^Foxp3^+^ regulatory T cells (Tregs), CD4^+^Foxp3^−^ effector T cells (Teffs), and CD8^+^ T cells in blood, lung, and kidney. (B) Representative FACS plots identifying four CD45^+^CD3^−^ non-T cell populations in blood, lung, and kidney, based on CD11b (monocyte marker) and CD11c (dendritic cell marker) expression. Bar graphs display the relative proportions of each subset (excluding double-negative cells) in ApoE^−/−^ and WT groups. (C) Representative histograms showing CD155 expression (mean fluorescence intensity, MFI) in three non-T cell subsets—CD11b^+^CD11c^−^ (CD11b single-positive, SP), CD11b^+^CD11c^+^ (double-positive, DP), and CD11b^−^CD11c^+^ (CD11c SP)—as well as in CD3^+^ T cells. (D) Quantitative analysis demonstrates that CD155 expressions in CD45^+^CD3^+^ T cells were reduced in ApoE^−/−^ blood, lung, and kidney compared with WT controls, whereas it was increased in CD45^+^CD3^−^ non-T cells in ApoE^−/−^ blood. (E) Bar graphs illustrating CD155 expression was modestly increased in CD11b^−^CD11c^−^ cells in blood but decreased in DP (CD11b^+^CD11c^+^) and CD11b^−^CD11c^−^ subsets in lung and kidney. (F) Expression of homeostatic immune markers CX3CR1, CD103, and CD206 was used to further define DP and CD11c SP populations in ApoE^−/−^ mice. CD155 expression was consistently lower in CD206^−^ than in CD206^+^ cells within both populations across all tissues. (G) In 12-wks-high-fat western diet fed ApoE^−/−^ mice (*n* = 9, male = 4, female = 5), CD155 expression levels were significantly lower in CD206^−^ cells compared with CD206^+^ cells in DP and CD11c SP subsets in Aorta. (H) Representative immunofluorescence images showing CD155, CD206, and CD68 staining in the aortic sinus of ApoE^−/−^ mice following 12 weeks of chow-diet. CD155 staining was detected in a subset of CD68^+^CD206^+^ cells surrounding the aortic sinus lesion, whereas additional punctate CD155 signals were observed within the plaque without clear colocalization with CD68. MFI, mean fluorescence intensity; WT, wild-type.

**TABLE 1 |:** Flow cytometry antibodies and reagents.

Antibody	Conjugation	Vendor	Catalog
CD45	PE	Biolegend	#103106
CD3	AF647	BD	#557869
CD3	BV650	Biolegend	#100229
CD11b	BV780	Biolegend	#101243
CD11c	BV605	Biolegend	#117334
CD103	PE-Cy7	Biolegend	#121426
CD206	BV421	Biolegend	#141717
CX3CR1	AF647	BD	#567805
TIGIT	BV421	Biolegend	#142111
CD4	APC-CY7	Biolegend	#100526
CD4	BUV395	BD	#740208
CD155	BUV395	BD	#743258
CD8a	APC-CY7	BD	#557654
Propidium iodide solution		Biolegend	#421301

## Data Availability

The data that support the findings of this study are available on request from the corresponding author. The data are not publicly available due to privacy or ethical restrictions.

## Abbreviations:

ApoE^−/−^: apolipoprotein E knockout mice
HF: high-fat diet
NC: normal chow
PBMC: peripheral blood mononuclear cells
Treg: CD4+Foxp3+ regulatory T cells
WT: wild type mice
